# Inflammatory Markers and Episodic Memory Functioning in Depressive Disorders

**DOI:** 10.3390/jcm11030693

**Published:** 2022-01-28

**Authors:** Katarzyna Wachowska, Janusz Szemraj, Janusz Śmigielski, Piotr Gałecki

**Affiliations:** 1 Department of Adult Psychiatry, Medical University of Lodz, 91-229 Lodz, Poland; piotr.galecki@umed.lodz.pl; 2 Department of Medical Biochemistry, Medical University of Lodz, 92-215 Lodz, Poland; janusz.szemraj@umed.lodz.pl; 3 Department of Health Sciences, State University of Applied Sciences in Konin, 62-510 Konin, Poland; janusz.smigielski.stat@gmail.com

**Keywords:** depression, inflammation, episodic memory

## Abstract

Depression is a psychiatric disorder that is observed to be associated with changes in levels of inflammatory markers and deterioration in cognitive functioning. Here, we combined the biochemical tests of IL-1 and IL-6 serum levels and the expressions of genes encoding these interleukins with cognitive assessment of episodic memories, and examined 50 depressed patients and 37 healthy participants. Results confirmed increased serum levels of IL-1 and IL-6 in the study group when compared to healthy volunteers. Moreover, episodic memory, in terms of answering structured questions (but not free recollection of past events) deteriorated among depressed patients. The described parameters neither correlated with each other nor with the two measures of severity of depression—HDRS score and years of psychiatric treatment. Although both observed dysfunctions—cognitive and immune—among depressed patients are confirmed, they do not seem to covary in the present study.

## 1. Introduction

More than 350 million people worldwide suffer from depression, making it one of the most common psychiatric disorders. Approximately, one in six people suffer from depression at some time in their life [1]. The disorder most often affects young people between the ages of 20 and 40. For individuals between 10 and 24 years of age, it is the tenth leading cause of DALYs (disability adjusted life-years), and for people between 25 and 49 years of age, it is the sixth leading cause of DALYs (according to GBD terminology, one DALY is defined as the loss of the equivalent of one full year of health) [2]. Depression may lead to many dangerous complications, including self-harming behaviors, the most lethal of which is suicide [2]. It also causes severe suffering, for the patients and their loved ones. Several studies discuss the increased activities in the immune systems of patients suffering from depression [3,4,5,6,7]. A large meta-analysis on the associations between inflammation and depression was conducted [5,6,8,9,10], and the results largely confirmed that relation. The most frequently analyzed peripheral markers of inflammation in the context of depression seem to be IL-1 and IL-6, although there are many others (pro- and anti-inflammatory) also being investigation [11]. IL-1 and IL-6 are pro-inflammatory cytokines.

Cognitive impairment is another important observation associated with depression. It occurs during the course of depressive disorders, in various domains of cognition—memory, attention, and executive functioning [12,13,14,15]. Cognitive deterioration has become such an important part of depression that it is listed as one of the criteria of the disease, according to ICD-11 and DSM 5 [16,17]. Moreover, a meta-analysis on the links between various cognitive domains and depression has been conducted [12,13,14], confirming the difficulties in cognitive functioning experienced by depressed patients.

We combined two important areas (cognition and immune function) of patients functioning, and researched their associations with depression (and with each other). In order to find a scientific basis for our research, we went through a professional database, searching for previously conducted studies. We limited the results to the last five years; still, we found many research studies. In a large scientific database, Google Scholar—searching for the phrase “IL 1 cognition depression”, with a time frame of 5 years (2016–2021) resulted in more than 91,000 records, whereas the phrase “IL 6 cognition depression” provided 18,000 records. Some records are studies that combined both peripheral markers of inflammation (IL-1 and IL-6) with depression [18], and some with just one peripheral marker. The overall impression is that this topic has been of high interest to scientists across the world. Basically, the results confirm the elevated levels of IL-1 and Il-6 as well as cognition impairment among patients with depression [18,19]. It has also been suggested in longitudinal studies that elevated levels of interleukins might precede mental disorders [19,20] and are associated with certain genetic variants [21]. It was a bit more difficult to find a correlation between the cognition and inflammation domains. However, in a number of research studies, IL-1β levels [18,22] and IL-6 [22,23,24] were negatively correlated with some areas of cognitive functioning. It has been suggested that this correlation depends on other variables as well [25]. It has also been noted that some therapeutic interventions, such as electroconvulsive therapy (ECT) and repetitive transcranial magnetic stimulation (rTMS), have possible neuromodulatory effects, and affect some domains of cognitive functioning [26,27]. That would explain their beneficial effects and provide background to plan further interventions. It has been suggested that special tools should be constructed to examine cognitive difficulties during depression [28]. Both cognitive dysfunction and inflammatory markers are associated with suicidal ideation; however, it has been difficult to confirm interactions between these two markers [29]. Moreover, inflammatory markers—IL-1, IL-6, and CRP—have been associated with the kynurenine pathway and activation of the hippocampus, but not with scores in memory testing [30].

The aim of the presented research was two-fold; first, to examine an example of a cognitive domain: functioning of memory of events, known as episodic memory, and two examples of inflammatory markers—IL-1 and IL-6—among patients suffering from depressive disorders; second, to compare the levels of these parameters with healthy volunteers.

## 2. Materials and Methods

The study involved 87 participants, male and female. Written, informed consent was obtained from all participants prior to the procedure. The study design was constructed with the principles of the Declaration of Helsinki, and it received approval from the Bioethics Committee at the Medical University of Lodz, consent number RNN/45/15/KE of 17 March 2015.

The group consisted of two subgroups: 50 were diagnosed with recurrent depressive disorders while 37 were healthy control subjects. A summary of the study group characteristics is presented in Table 1. A summary of the healthy control group is presented in Table 2.

All participants were Poles from the central part of Poland. Participants were included in the study on the basis of the diagnosis of depressive disorders according to ICD-10 and DSM 5 classifications. Patients with co-morbid substance abuse (apart from tobacco), severe head trauma in past medical history, and diagnosed dementia were excluded from the study.

Severity of depression assessment. The Hamilton Rating Depression Scale (HDRS), version 17, was used to objectify the severity of depressive symptoms at the time of the study. The scale was completed by the researcher (psychologist) during each interview.

Memory assessment. The episodic memory questionnaire was developed on the basis of the existing literature, autobiographical memory questionnaires, and the researcher’s own concepts. It was checked by three independent psychologists—competent judges. It consisted of three tasks. Task 1 was to fill out the annual dates in the table, covering the events from social life. This task score was named “a number of social life events”. Task 2 was the "lifeline" in which the examined person was asked to provide short descriptions of events from their life, dated annually—an example of free recollections of events from one’s own past, put chronologically. To objectify the score (more years of life might cause more memories; therefore, older participants might have received more points), the number of memories was counted, and then the number of events per year of life was converted into an indicator (number of memories on the lifeline/age of the examined person). Task 3 was a set of questions in the form of a structured interview, covering various aspects and periods of the respondent’s life (name, surname, date of birth, data on family issues, circumstances of acquiring skills, data on education, memories of toys, and others). Each question score was based on the amount of information provided by the examined person. The total score of Task 3 was named “total score episodic memory”. The examined person performed the test on his/her own after reading the instructions and after a short introduction by the examiner. The personal questionnaire prepared by researchers was also used, covering basic sociometric data, the current course of the disease, and previous psychiatric treatments.

Biochemical methods. A sample of blood (10 mL) was taken from participants who gave informed consent for blood tests (50 in the depressed group, 30 in the healthy control group). 

Determination of protein expression/determining protein concentration. A total of 150 µL of the reaction mixture was added to pits containing 150 µL of serum, diluted 10 times in 10 mM of phosphate buffered saline, pH 7.4, and incubated (2 h, 37 °C). In order to specify protein concentration, an analytical curve for serum albumin was determined. Both the examined samples and the reference samples were made parallel in three repetitions. Sample absorbance was measured using Multiskan Ascent Microplate Photometer (Thermo Labsystems) at λ = 562 nm, and the total protein concentration was calculated from the standard curve equation.

Enzyme-linked immunosorbent assay (ELISA). The concentration of proteins IL1β and IL6 in the serums of the patients were determined using Human IL1β and IL6 Elisa Kit (R D Systems, Minneapolis, MN, USA), according to the protocols provided by the manufacturer. β-actin was used for endogenous control of protein concentration in the samples and determined with the help of Human Actin Beta (ACTB) ELISA Kit (BMASSAY, Beijing, China) based on the manufacturer’s recommendations. A total of 100 μL of serum (ρprotein = 0.5 mg/mL) was added to pits coated with antibodies specific for the analyzed proteins, and incubated (1.5 h, 37 °C). The content was removed, and the pits were rinsed three times in 10 mM of phosphate buffered saline and incubated (1 h, 37 °C) with 100 μL of biotinylated antibodies specific for the analyzed proteins. Then, the content was removed, and the pits were rinsed three times in 10 mM of phosphate buffered saline and incubated (30 min, 37 °C) with 100 μL of ABC Working Solution. The content was removed, and the pits were rinsed five times in 10 mM of phosphate buffered saline and incubated (10 min, 37 °C) with 90 μL of TMB substrate. After adding 100 μL of TMB Stop Solution, the absorbance of the samples was measured using Multiskan Ascent Microplate Photometer (Thermo Labsystems) at λ = 450 nm. In order to determine protein concentration, analytical curves for the analyzed proteins were made.

Statistical analysis. The data were verified for normality of distribution and equality of variances. Correlations between the results of biochemical tests, cognitive tests, and HDRS were analyzed with the Spearman’s rank correlation coefficient. Gender structure analysis was performed by the Chi-square test. The Mann–Whitney U test was used to compare the average values received. The statistical analysis was performed using the Statistica® 13.1 CSS program (StatSoft Polska, u. Kraszewskiego 36 30-110 Kraków). The results of the quantitative variables are presented as mean ± standard deviation (SD), median. The limit of statistical significance was set at *p* < 0.05 for all analyses.

## 3. Results

### 3.1. Inflammatory Markers

Results obtained from the biochemical analysis of blood samples in the study group and the control group are presented in Table 3 and Table 4, respectively.

Statistical analysis of the variables was performed with the Mann–Whitney U test to compare the average values received. The results are presented in Table 5.

**Figure 1:**
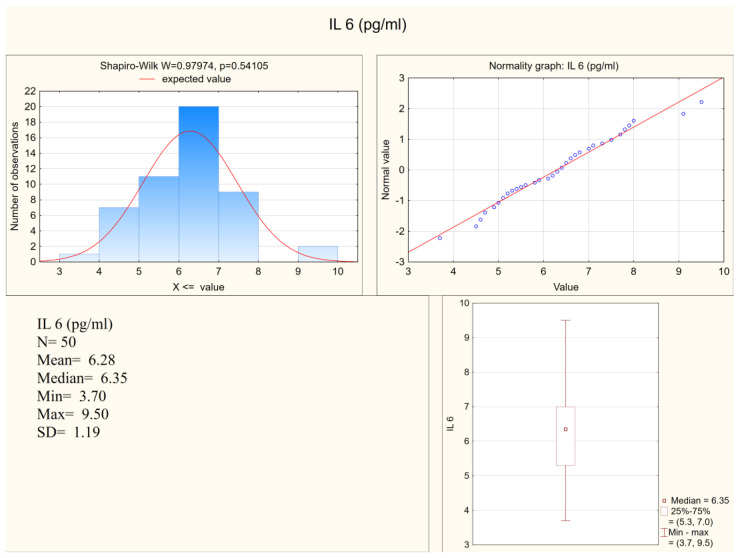
IL-6 in the study group.

**Figure 2:**
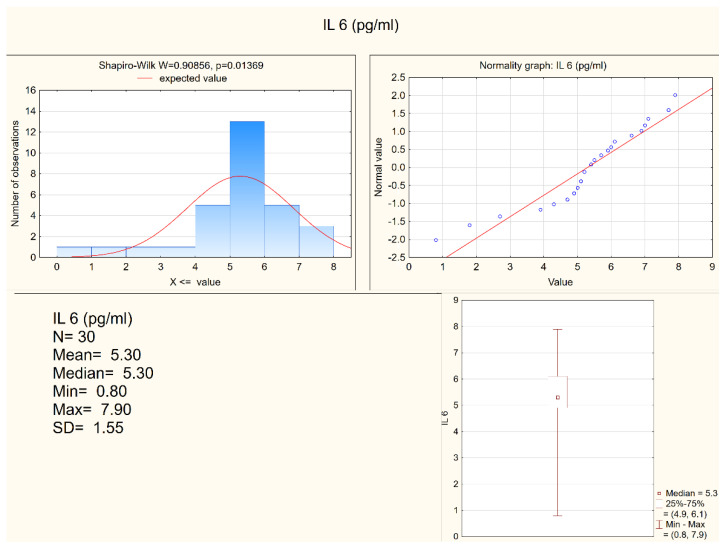
IL-6 in the control group.

**Figure 3:**
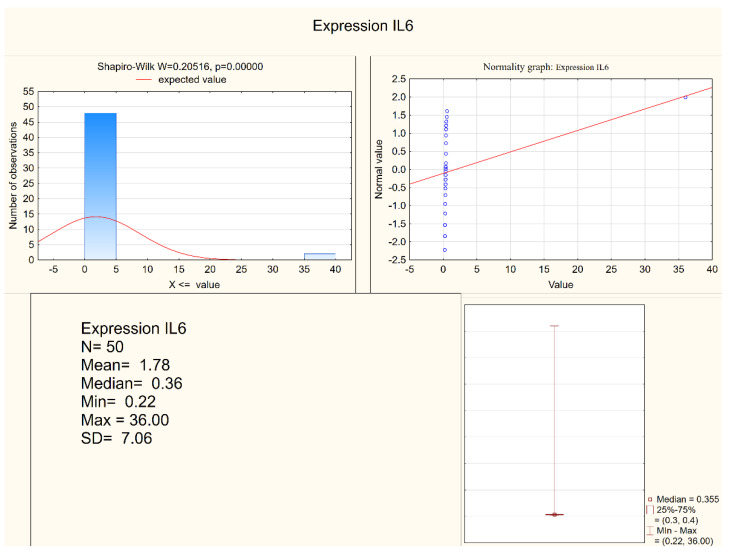
Expression of IL-6 in the study group.

**Figure 4:**
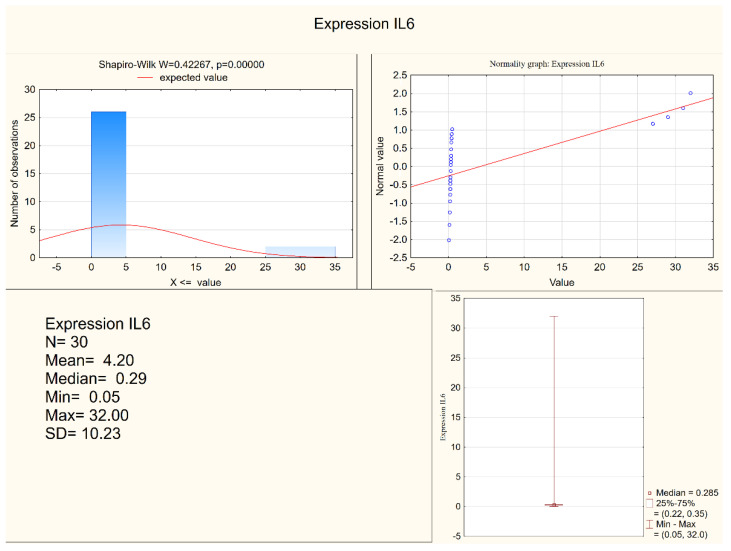
Expression of IL-6 in the control group.

Statistically significant differences between the depressed patients and the healthy controls were observed in the levels of IL-1 β, IL-6, and in the expression of IL-6, but not in the expression of IL-1β. Both IL-1 β and IL-6 serum levels were higher in the depressed group (mean levels 10.75 pg/mL and 6.28 pg/mL, respectively) when compared to the control group (mean levels 8.28 pg/mL and 5.3 pg/mL, respectively). Figure 1 and Figure 2 present IL-6 (pg/mL) in the study group and the control group (respectively). Alternatively, measures of expression of IL-1β and IL-6 were higher in the control group (mean levels 5.31 and 4.20, respectively) when compared to the study group (mean levels 2.47 and 1.78, respectively), although they were only statistically significant in the IL-6 expression level. Figure 3 and Figure 4 present expressions of IL-6 in the study group and the control group (respectively).

### 3.2. Memory Function

Results obtained from the psychological tests in the study group and control group are presented in Table 6 and Table 7, respectively.

Statistical analysis of the variables was performed with the Mann-Whitney U test to compare the average values received. The results are presented in Table 8.

Statistically significant differences between the depressed patients and healthy controls were observed in the total results obtained in the episodic memory questionnaire (total score). The healthy controls obtained more points in that part of the test, which means they were able to answer more questions in a more detailed way than the depressed patients. Although higher in the healthy control group, neither the number of memories of social events nor the indicator was different with the statistical significance between the compared groups.

### 3.3. Correlations Coefficients among Serum Levels of Interleukins and Results in Memory Tests and Depression Severity

Correlations between serum levels of interleukins (IL-1β, IL-6), levels of their expressions, and results in the memory test (total score, number of events on a lifeline, number of memories converted to the number of years “indicator”) were analyzed with Spearman’s rank correlation coefficient. Correlations were performed for each group separately. Parameters chosen as measures of severity of depression (HDRS score, years of psychiatric treatment) were also analyzed (only in the study group). The results are presented in Table 9 and Table 10.

Reverse correlation on a statistically significant level was observed for the IL-6 serum level and the number of events on a lifeline in the control group. Other parameters were not associated at the statistical level, allowing for inference.

## 4. Discussion

The presented study confirmed previous observations of increased inflammatory markers among patients suffering from depression [3,4,5,6]. Serum levels of IL-1 β and IL-6 were higher among the depressed patients than healthy controls with statistically significant differences. When it comes to expression of genes encoding interleukins 1β and 6, those levels were higher in the healthy control group. However, the difference was statistically significant only when IL-6 expression was analyzed. Scientific understanding of depression is now significantly based on the biological model of chronic inflammation. However, well confirmed observations of raised inflammatory markers among depressed patients did not meet satisfactory explanation of the mechanism of this phenomenon [7]. The aim of the study was to search for the association between inflammation and episodic memory function in depressed patients.

Overall comparison of the results in an episodic memory test suggests that, in all parameters, the healthy participants achieved higher scores than the depressed group. However, those results mostly did not reach a statistically significant level, therefore inhabiting authors from making conclusions. The episodic memory function was different with statistical difference only in one of three measured parameters—total score—counted on the basis of structured interview. Patients with depression did not give answers at all or gave less detailed information when answering standard questions about their pasts. When it comes to scores in the number of remembered social events and the number of events on a lifeline—there were no statistically significant differences between the groups.

Although extensive statistical analyses on correlations between the examined parameters were performed in both the study and the control group, they failed to confirm that the levels of inflammatory markers and tested memory functions were associated. It was only among the healthy control group where an inverse correlation was noted between the IL-6 level and the number of events put on lifeline. However, the result was not repeated for the indicator, which is a more objective measurement. The severity of depression measured in years of treatment and the HDRS score were also not associated with levels of interleukins.

Memory processes are broad concepts which include the phases of recording, storing, and reproducing an experience. This research focused only on one domain of memory, in which the material concerning an individual’s life history was stored, i.e., episodic memory. Its proper functioning enables the formation of one’s sense of identity, building a sense of self-coherence over time, a sense of continuation, and stabilization [31,32]. Research on the functioning of autobiographical memories in patients with depression has so far focused largely on qualitative assessment. Brittlebank et al. (1993) conducted a study on the emotional signs of memories and the tendency—in patients with depression—to over-generalize autobiographical material. The functioning of patients with depression, in terms of autobiographical memory, is characterized by (when compared to healthy people) a tendency to recall negative emotional events more quickly, and a tendency to recall (in response to keywords) and over-generalize memories, rather than specific ones [33].

In the presented study, we approached episodic memory in a quantitative way. Emotional input of memories was not considered. The participants were asked to freely recall memories and put them on a lifeline, as well as to answer structured, direct questions about their pasts. There was a difference between the results obtained in those two ways—lower results among the depressed subjects when compared to the healthy control subjects. However, a statistical analysis showed that the depressed subjects scored “definitely” worse in recalling information when directly asked about it. Therefore, free recollections of events did not diminish among depressed patients when compared to healthy volunteers on a level of statistical significance. The reasons for the observed phenomena are unknown. Wesnes et al. (2016) described a study linking the level of memory processes functioning with the 5-HT1A receptor genotype. They showed that, in the group of patients suffering from depression, a specific genotype of receptor polymorphism is associated with better maintenance and retrieval of information from episodic and working memory [34]. A meta-analysis of global reports that focused on cognitive functioning of patients with depression showed that intensification of depressive symptoms negatively affects cognitive functioning in areas of episodic memory, executive functions, and data processing speed [12,13,14,15]. The presented research failed to confirm associations between level of inflammation (measured by peripheral concentrations of inflammatory markers) and functioning of episodic memory. Another possible explanation of memory impairment might be organic changes in the brain caused by chronic inflammation, due to hyperactivity of HPA axis. It was observed that the hippocampus—the region largely responsible for memory functioning—is reduced among depressed subjects [35], and its activation seems to be attenuated in imaging research [36]. Both of these studies conclude that repeated episodes of depression might negatively affect this part of the brain. It was also established that an elevated level of the peripheral inflammatory marker—IL-6—covaries inversely with hippocampal grey matter [37]. Therefore, it is possible that memory dysfunction is caused by neurodegeneration process caused by chronic inflammation, both central and peripheral, observed among depressed patients. Thus, measurements of cognition might not be directly related to levels of inflammation, but more to its persistence. Inflammatory markers, such as TNFα, tend to affect endothelial cells, building the blood–brain barrier as well as the structure of brain parenchyma, including that situated in the region of the hippocampus [4]. Moreover, cells of microglia are part of the neuroinflammatory process and influence a number of neurons and the synaptic structure [38,39]. It was also suggested that early stages of development, of both the nervous and immune systems, might be crucial in future mental problems [40]. This subject needs more research, especially studies conducted with interdisciplinary co-operation, including psychiatrists, psychologists, and specialists in the field of radiology.

## 5. Conclusions

Several conclusions based on the described research might be formed:

1. Increased serum levels of inflammatory markers IL-1 and IL-6 are observed among patients suffering from depression when compared to healthy controls.
2. Episodic memory functioning, in terms of answering structured questions about the past, is deteriorated, in comparison to healthy control groups.
3. Episodic memory functioning, in terms of free recalling of events, is not different when compared to healthy participants.
4. Although the presented data confirm previous observations of increased levels of inflammatory markers among depressed patients, the collected data do not allow associating inflammatory imbalance with episodic memory functioning. The observed memory disturbances are not associated with changes in serum levels of examined inflammatory markers in a study group. However, in the healthy control, group we observed a reverse correlation between the serum level of IL-6 and free recalling of life events (only for a number of remembered events, not for an objective indicator). This might suggest that non-depressed subjects have more difficulties with free recollection of memories when there is a biochemical sign of an inflammatory process.
5. The presented study has limitations, such as a relatively low number of participants, as well as of the control group, and a lack of prospective observations of depressed patients. Moreover, examining memories of life events is quite difficult since it is a strongly subjective matter. Therefore, the obtained results need to be treated with caution and should be confirmed in further research.

## Figures and Tables

**Table 1:** Demographic characteristics of the study group. Parameters associated with severity of depression.

Demographic Characteristics	Study Group
Number of patients	50
Age	46.34 ± 11.18
Number of hospitalizations	2.06 ± 2.11
Male/Female	40/10
Living in a big city/small city	34/16
Smokers/non-smokers	32/18
Education secondary education level/higher level	39/11
**Parameters Associated with Severity of Depression**	
Years of psychiatric treatment	4.73 ± 6.25
Result in HDRS	18.27 ± 7.2

**Table 2:** Demographic characteristics of healthy control group.

Demographic Characteristics	Study Group
Number of participants	37
Age	40.2 ± 16.42
Male/Female	28/9
Living in a big city/small city	30/7
Smokers/non-smokers	8/29
Education secondary education level/higher level	11/26

**Table 3:** Inflammatory markers in the study group.

Name of a Marker	Medium	Median	Min	Max	SD
IL-1 β (pg/mL)	10.75	11.0	7.8	13.2	1.45
Expression IL1 β	2.47	0.73	0.47	88.0	12.34
Il-6 (pg/mL)	6.28	6.35	3.7	9.5	1.18
Expression IL6	1.78	0.36	0.22	36.0	7.06

**Table 4:** Inflammatory markers in control group.

Name of a Marker	Medium	Median	Min	Max	SD
IL-1 (pg/mL)	8.28	8.05	4.80	11.10	1.38
Expression IL1 β	5.31	0.67	0.29	69.00	16.95
Il-6 (pg/mL)	5.30	5.30	0.80	7.90	1.54
Expression IL6	4.20	0.29	0.05	32.00	10.22

**Table 5:** Inflammatory markers—comparison between groups.

Name of a Marker	N	Mean	Med	Min	Max	SD	Skewness	Mann-Whitney U Test	*p* Value
IL-1 (pg/mL)	80.00	9.83	9.80	4.80	13.20	1.86	−0.20	5.77	0.00
Expression IL1 β	80.00	3.53	0.70	0.29	88.00	14.21	5.09	1.89	0.06
Il-6 (pg/mL)	80.00	5.91	5.95	0.80	9.50	1.40	−0.62	2.76	0.01
Expression IL6 β	80.00	2.69	0.33	0.05	36.00	8.40	3.36	2.88	0.00

**Table 6:** Memory function in the study group.

Name of a Parameter	Medium	Median	Min	Max	SD
Task 1 Memories of social events	6.93	7.00	0.00	25.00	5.32
Task 2 Indicator	0.40	0.32	0.00	1.73	0.31
Total score episodic memory	22.02	22.00	0.00	33.00	7.17

**Table 7:** Memory function in the control group.

Name of a Parameter	Medium	Median	Min	Max	SD
Task 1 Number of memories of social events	7.16	7.00	0.00	37.00	6.31
Task 2 Indicator	0.51	0.47	0.00	1.21	0.33
Total score episodic memory	27.54	27.00	16.00	35.00	4.11

**Table 8:** Memory function comparison between groups.

Memory Parameter	N	Mean	Med	Min	Max	SD	Skewness	Mann–Whitney U Test	*p* Value
Task 1 Number of memories of social events	80.00	7.04	7.00	0.00	37.00	5.77	2.11	0.19	0.85
Task 2 Indicator	87.00	0.45	0.39	0.00	1.73	0.32	1.31	−1.86	0.06
Total score episodic memory	87.00	24.37	25.00	0.00	35.00	6.63	−0.96	−3.78	0.00

**Table 9:** Correlation coefficients among serum levels of interleukins and results in memory tests and depression severity—study group.

Study Group	Spearman’s Rank Order Correlation MD Removed in Pairs The Marked Correlation Coefficients Are Significant *p* < 0.05000			
	N	R	*t*-Student Test *t* (N-2)	*p*
IL 1β (pg/mL) and HDRS score	48	−0.136	−0.934	0.355
IL 1 β (pg/mL) and total score episodic memory	50	0.203	1.436	0.158
IL 1 β (pg/mL) and years of psychiatric treatment	45	−0.045	−0.293	0.771
IL 1 β (pg/mL) and number of events on “lifeline”	50	0.026	0.179	0.859
IL 1 β (pg/mL) and indicator	50	0.085	0.593	0.556
Expression IL1 β and HDRS score	48	0.076	0.516	0.608
Expression IL1 β and total score episodic memory	50	0.037	0.254	0.801
Expression IL1 β and years of psychiatric treatment	45	0.152	1.007	0.320
Expression IL1 β and number of events on “lifeline”	50	−0.152	−1.068	0.291
Expression IL1 β and indicator	50	−0.050	−0.347	0.730
IL 6 and HDRS score	48	−0.136	−0.930	0.357
IL 6 and total score episodic memory	50	0.047	0.323	0.748
IL 6 and years of psychiatric treatment	45	0.225	1.516	0.137
IL 6 and number of events on “lifeline”	50	−0.111	−0.774	0.443
IL 6 and indicator	50	−0.021	−0.148	0.883
Expression IL6 and HDRS score	48	−0.244	−1.708	0.094
Expression IL6 and total score episodic memory	50	−0.097	−0.672	0.505
Expression IL6 and years of psychiatric treatment	45	0.029	0.191	0.849
Expression IL6 and number of events on “lifeline”	50	0.013	0.088	0.931
Expression IL6 and indicator	50	0.099	0.691	0.493

**Table 10:** Correlations coefficients among serum levels of interleukins and results in memory tests and depression severity—healthy control group.

Healthy Control Group	Spearman’s Rank Order Correlation MD Removed in Pairs The Marked Correlation Coefficients Are Significant *p* < 0.05000			
	N	R	*t*-Student Test	*p*
IL 1 β (pg/mL) and total score episodic memory	30	−0.079	−0.421	0.677
IL 1 β (pg/mL) and number of events on “lifeline”	30	−0.062	−0.326	0.747
IL 1 β (pg/mL) and indicator	30	−0.002	−0.009	0.993
Expression IL1 β and total score episodic memory	30	0.276	1.521	0.139
Expression IL1 β and number of events on “lifeline”	30	0.051	0.268	0.791
Expression IL1 β and indicator	30	0.238	1.294	0.206
IL 6 and total score episodic memory	30	−0.104	−0.554	0.584
IL 6 and number of events on “lifeline”	30	−0.457	−2.718	0.011
IL 6 and indicator	30	−0.360	−2.045	0.050
Expression IL6 and total score episodic memory	30	−0.129	−0.686	0.498
Expression IL6 and n number of events on “lifeline”	30	0.112	0.594	0.557
Expression IL6 and indicator	30	0.047	0.251	0.803
